# Systematic review of point-of-need molecular diagnostics for rotavirus and enteric adenoviruses F40/F41

**DOI:** 10.1186/s12879-026-13081-4

**Published:** 2026-03-13

**Authors:** Fatou Kiné Top, Cheikh Tidiane Diagne, Cheikh Talibouya Touré, Julie-Melissa Gonfouli, Oumar Faye, Yakhya Dieye, Julien Reboud, Abdourahmane Sow, Jonathan M. Cooper, Martin Faye

**Affiliations:** 1 Virology Department, Institut Pasteur de Dakar, 36 Avenue Pasteur, BP 220, Dakar, Senegal; 2 Diatropix Department, Institut Pasteur de Dakar, 36 Avenue Pasteur, BP 220, Dakar, Senegal; 3 Microbiology Department, Institut Pasteur de Dakar, 36 Avenue Pasteur, BP 220, Dakar, Senegal; 4 Public Health Department, Institut Pasteur de Dakar, 36 Avenue Pasteur, BP 220, Dakar, Senegal; 5 University of Glasgow, Glasgow, Great Britain, Scotland

**Keywords:** Gastroenteritis, Rotavirus, Enteric adenoviruses F40/F41, Molecular diagnostics, Point-of-care

## Abstract

**Background:**

Diarrhea is a leading cause of mortality in children under five, with rotavirus and enteric adenoviruses F40/F41 being significant contributors. In regions like Africa and Southeast Asia, the incidence is exacerbated by poor hygiene and sanitation, as well as the challenges around availability and affordability of healthcare and disease diagnostics. The objective of this systematic review was to identify and evaluate molecular assays for the detection of rotavirus and enteric adenoviruses F40/F41, with particular emphasis on diagnostic performance and suitability for point-of-care use.

**Methods:**

A systematic review of literature from 2001 to 2024 was conducted following PRISMA guidelines. Out of 659,613 records screened, a subset of primary studies reporting on molecular assays development or diagnostic performance met the inclusion criteria. Studies exclusively focused on epidemiology, vaccine impact, or narrative reviews were excluded from the analytical synthesis.

**Results:**

The review identified 26 PCR-based assays and 3 isothermal amplification assays. Reported clinical sensitivities ranged from approximately 85% to 100%, with specificities generally above 95% across studies. Several assays also demonstrated low limits of detection and turnaround times compatible with decentralized use. In particular, three LAMP assays and three PCR-based methods fulfilled key performance and operational criteria for point-of-care diagnostics. The review concluded that molecular diagnostics, especially LAMP assays, are effective for rapid detection of rotavirus and enteric adenoviruses F40/F41.

**Conclusion:**

While PCR assays are considered the gold standard, LAMP assays are more accessible and faster, making them better suited for use in resource-limited settings. These assays show promising performance and may support point-of-care diagnostics.

**Supplementary Information:**

The online version contains supplementary material available at 10.1186/s12879-026-13081-4.

## Introduction

Acute gastroenteritis remains a major public health problem, particularly among children under five years of age. It is caused by a wide range of pathogens, including bacteria, parasites, and viruses. Among these etiological agents, viruses are responsible for a substantial proportion of both endemic and epidemic gastroenteritis worldwide, with rotavirus and human adenovirus (HAdV) among the main causative agents [1]. According to the World Health Organization (WHO), more than 70% of diarrhea-related deaths in children under five occur in Africa and Southeast Asia [2]. The incidence of infectious diarrhea is higher in low- and middle-income countries than in high-resource nations, due to poor hygiene, unsafe water, contaminated food and inadequate disposal of waste and faeces [3].

Rotavirus infection is the most common cause of severe gastroenteritis in infants and young children worldwide, with group A the most common [4] while HAdV species F (types 40 and 41) are linked to gastrointestinal diseases and are responsible for up to 20% of diarrhea episodes worldwide [5]. Rotaviruses are characterized by extensive genetic diversity which underscores the importance of sensitive and broadly reactive molecular diagnostic tools capable of detecting both common and emerging rotavirus genotypes in clinical and surveillance settings [6].

In 2008, five countries accounted for more than half of the 450,000 deaths due to rotavirus diarrhea, including three African countries: The Democratic Republic of Congo, Ethiopia and Nigeria. In Ethiopia, a meta-analysis (12 studies, children under 5 years) reported a pooled rotavirus prevalence of 23% (95% IC: 22–24%) [7]. In Nigeria, a nationwide meta-analysis (1982–2021) estimated a similar average rotavirus prevalence of 23% (95% IC: 19–27%) among children under five years of age with acute gastroenteritis. The prevalence was slightly higher in the southern region (27%) compared to the northern region (20%) [8]. In the Democratic Republic of Congo, group A rotaviruses were detected in 76% of stool samples analyzed during epidemics of acute gastroenteritis in children under 5 living in Kinshasa. Other countries also have relatively high prevalence. For example, in Cameroon, rotavirus was detected in 28.5% to 42.0% of diarrheal samples; infection occurs throughout the year, and is most frequent in children under five. In Gabon, a high prevalence of enteric viruses was detected in children, with 60.9% of stool samples positive for at least one enteric virus. In Senegal, a study of community-acquired infectious diarrhea in children under five showed that rotavirus was the most commonly detected enteric pathogen in younger patients (< 24 months) [9]. Although rotavirus infection is widespread worldwide, most deaths occur in developing countries. By the age of 3–5, it infects almost all children in all settings. Neonatal infections do occur; however, they are often asymptomatic or mild, probably due to maternal protection by antibodies. Clinical disease is most common in children aged between 4 and 23 months, who are at high risk of developing severe disease requiring hospitalization [10].

Adenoviruses have also been associated with 3.1–13.5% of paediatric diarrhea cases in studies carried out in Europe, Asia, North and South America; types 40 and 41 are thought to comprise between 37.5 and 100% of these adenoviruses. Epidemiological studies in South Africa have shown a prevalence of enteric adenoviruses similar to that reported worldwide [11]. HAdV-induced acute gastroenteritis is transmitted mainly via the feco-oral route and is associated with prolonged diarrhea, which can lead to dehydration and malnutrition in infants [12].

Although these viruses are primarily transmitted through the feco-oral route and contaminated environments, many low-resource settings face significant challenges related to limited access to laboratory diagnostics, inadequate surveillance systems, and constrained healthcare infrastructure, which hinder timely detection and management of viral gastroenteritis [13]. Consequently, it is advisable to rapidly determine rotavirus and HAdV antigens in stool samples using immunochromatographic tests [14]. The recent application of quantitative molecular diagnostics to etiological studies of diarrhea has revealed increased estimates of diarrhea due to enteric adenoviruses. In a re-analysis of the Global Enteric Multicenter Study using quantitative polymerase chain reaction (*q*PCR) for a wide range of pathogens, adenoviruses F40/F41 were the second highest cause of diarrhea in infants after rotavirus [15]. Although rotavirus remains the most prevalent etiology of pediatric gastroenteritis, the clinical overlap with other enteric pathogens means that presumptive treatment based on prevalence alone risks mismanagement, delayed outbreak control, and missed surveillance opportunities. Rapid molecular diagnosis remains essential for accurate case differentiation and optimal patient outcomes.

The objective of this systematic review was to identify and critically evaluate molecular assays developed for the detection of rotavirus and enteric adenoviruses F40/F41. The review specifically aimed to: (i) summarize the types of molecular assays developed, (ii) assess their reported analytical and diagnostic performance, and (iii) identify assays with characteristics suitable for point-of-care use. These characteristics are particularly important in low-resource settings where access to centralized laboratories is limited.

## Methods

### Literature search strategy

We carried out a systematic review of the literature to identify studies on the development of molecular diagnostic assays for the detection of rotavirus and enteric adenovirus F40/F41. A systematic literature search was conducted in PubMed Central (PMC), ScienceDirect, and Google Scholar to identify studies reporting on molecular diagnostic assays for rotavirus and/or enteric adenoviruses F40/F41. Searches were performed for articles published between 2001 and 2024. Database-specific search strings were constructed using combinations of Medical Subject Headings (MeSH) terms and free-text keywords related to the pathogens and diagnostic methods. The core search concepts included: (i) rotavirus or enteric adenovirus F40/F41, (ii) molecular diagnostics, and (iii) point-of-care testing.

An example of the PubMed Central search strategy was: *(“Rotavirus” OR “Enteric adenovirus” OR “Adenovirus 40” OR “Adenovirus 41”) AND (“PCR” OR “RT-PCR” OR “LAMP” OR “isothermal amplification” OR “molecular diagnostic”) AND (“point-of-care”) *. Similar keyword combinations, adapted to the syntax of each database, were applied for ScienceDirect and Google Scholar.

This review was carried out in accordance with the established “preferred reporting elements for systematic reviews and meta-analyses” (PRISMA) protocol [16].

### Study screening and selection process

The initial search yielded a high number of records (*n* = 659,613), largely driven by broad keyword matching in Google Scholar and ScienceDirect. To ensure feasibility and relevance, several filtering steps were applied prior to manual screening. First, automatic database filters were used to restrict results to journal articles, excluding patents, conference abstracts, theses, and non-scientific documents. Second, title-based relevance filtering was applied to exclude records unrelated to viral gastroenteritis diagnostics (e.g., environmental virology, oncology-related adenovirus studies, or non-diagnostic molecular biology research). Duplicate records across databases were removed prior to screening.

Following this initial reduction, titles and abstracts were screened manually by two authors to assess eligibility based on predefined inclusion and exclusion criteria. Only studies reporting original data on molecular assay development or diagnostic performance were retained for full-text review. Discrepancies were resolved by consensus.

### Inclusion and exclusion of studies

In this review, molecular assays were classified as point-of-care (POC) if they met predefined operational criteria in addition to acceptable diagnostic performance. The criteria were based on commonly used definitions in global health and diagnostic literature and included: (i) rapid turnaround time (generally ≤ 60 min), (ii) minimal or no requirement for complex laboratory infrastructure, (iii) ease of use with limited technical training, and (iv) suitability for decentralized or near-patient testing environments. Assays meeting most of these operational characteristics, particularly those using isothermal amplification or integrated cartridge-based platforms, were considered compatible with POC applications.

We only included studies that were full texts reported in English on rotavirus or enteric adenovirus F40/F41 infection that met the case definition described by the WHO [17]. In short, they should have recruited cases of acute watery diarrhea, defined over a 48-hour period. We included primary research studies that reported on the development, analytical validation, or diagnostic performance evaluation of molecular assays for the detection of rotavirus and/or enteric adenoviruses F40/F41 in stool samples. Eligible studies were required to report at least one diagnostic performance parameter, including analytical sensitivity (limit of detection), clinical sensitivity, specificity, or time to result.

We excluded studies that were purely epidemiological (seroprevalence or surveillance), vaccine impact assessments, narrative reviews, meta-analyses, or studies not reporting original diagnostic performance data. Studies using only antigen detection methods (EIA/ELISA), electron microscopy, or lacking sufficient methodological detail were also excluded. Following refinement of the inclusion and exclusion criteria to focus exclusively on molecular assay development and diagnostic performance evaluation, the study selection process was repeated to ensure full consistency with the objectives of the review.

### Data extraction and analysis

Data were collected from included studies into a Microsoft Office Excel 2019 database (as described in Tables 1 and 2). This was designed based on performance characteristics required for tests developed by laboratories, as set out in the revised Clinical Laboratory Improvement Amendments (CLIA) [18] (analytical performance characteristics). To avoid confusion, each study was assigned a number and the following information was collected: author’s contact details, year of publication, country where the study took place, study duration/period, test methods (and their performance), age range, study setting. The technical characteristics of the assays (primer sequences, target region, incubation temperature) were also collected.

### Statistical analysis

Tables were generated using the **Microsoft Excel 2019 program**, and figures using **RStudio version 4.4.2**. As the included studies were heterogeneous in design, targets, and reported outcomes, no statistical meta-analysis was performed, and findings were synthesized qualitatively.

## Results

### Study selection

**Fig. 1:**
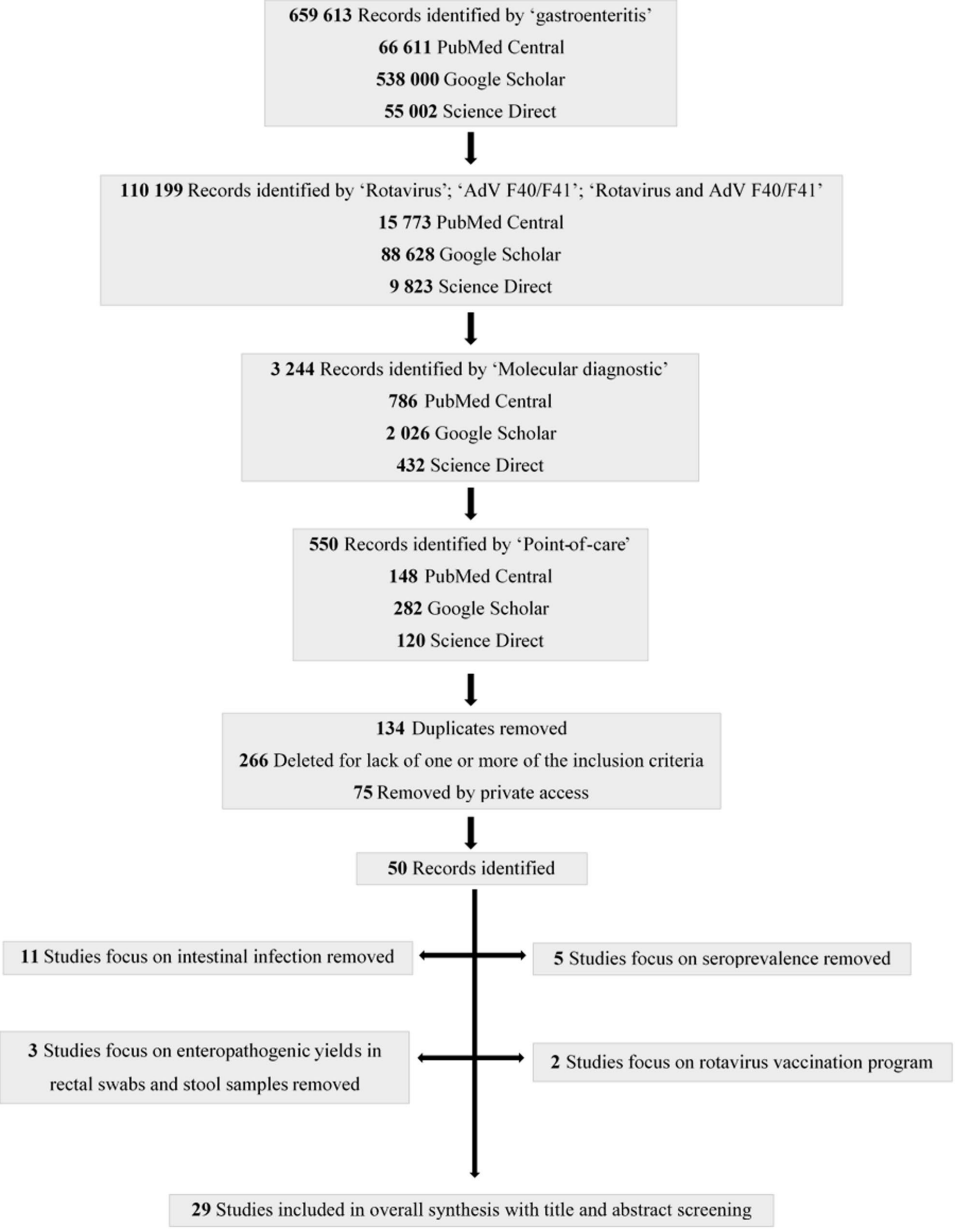
Flow-chart of the study selection process. Remark: Private for research articles = not publicly available. The article exists, but it is behind a paywall, or it is shared only with invited users, or the journal database requires institutional credentials to view or download the full text

The database search initially retrieved 659,613 records. After automated filtering, duplicate removal, and title-based relevance screening, a substantially reduced subset of records was subjected to manual title and abstract screening, resulting in 29 full-text articles assessed for eligibility (Fig. 1). After full-text screening, only studies reporting original data on molecular assay development or diagnostic performance were retained for qualitative synthesis. Epidemiological, seroprevalence, vaccine impact, and review studies were excluded from the analytical dataset and were used solely for contextual background. Only assays fulfilling the predefined operational criteria for point-of-care use, including rapid turnaround time and minimal equipment requirements, were classified as suitable for decentralized diagnostic settings.

### Descriptive characteristics of included studies

Descriptive characteristics were collected concerning the region, age group, study type, and study period. The included studies were mainly conducted in Europe, North America, and Asia, with fewer studies from Africa. Most articles focused on test development, particularly in Asian and European countries such as China, India, Japan, and several European nations, reflecting efforts to design rapid and accessible molecular diagnostic tools [19]. In contrast, diagnostic performance studies were more common in North America and Europe, often evaluating commercial multiplex assays in clinical settings. Regarding the study population, a large proportion of studies targeted children, the group most affected by viral gastroenteritis, while some performance studies included all age groups. Overall, the studies published between 2001 and 2024 show a shift from conventional PCR to real-time PCR, multiplex assays, and more recent isothermal amplification methods (Table 1).

**Table 1:** Characteristics of included primary studies evaluating molecular diagnostic assays for rotavirus and enteric adenoviruses F40/F41

Study type	Authors	Year	Study setting	Location	Age group	References
Development	Allard et al.	2001	Laboratory-based	Sweden	Not specified	[20]
	Avellón et al.	2001	Laboratory-based	Spain	Not specified	[21]
	Heim et al.	2003	Laboratory-based	Germany	Not specified	[22]
	Pang et al.	2004	Hospital laboratory	Canada	Children	[23]
	Logan et al.	2006	Hospital-based	Ireland	Children	[24]
	Gutiérrez-Aguirre et al.	2008	Hospital laboratory	Slovenia	Children	[25]
	Van Maarseveen et al.	2010	Clinical laboratory	Netherlands	All ages	[26]
	Nordgren et al.	2010	Hospital-based	Sweden	Children	[27]
	Khamrin et al.	2011	Hospital-based	Thailand	Children	[28]
	Liu et al.	2011	Hospital-based	Tanzania	Children	[29]
	Liu et al.	2012	Clinical laboratory	China	All ages	[30]
	Liu et al.	2013	Hospital-based	Tanzania	Children (< 5 years)	[31]
	Pang et al.	2014	Clinical laboratory	Canada	All ages	[32]
	Bennett et al.	2017	Clinical laboratory	UK	All ages	[33]
	Thongprachum et al.	2017	Hospital-based	Thailand	Children	[34]
	Kowada et al.	2018	Hospital-based	Japan	All ages	[35]
	Ye et al.	2018	Laboratory/POC prototype	China	Children	[36]
	Mitra et al.	2020	Hospital-based	India	Children	[37]
	Shuryaeva et al.	2024	Hospital laboratory	Russia	Children	[38]
Development + performance	Ziros et al.	2015	Laboratory and environmental samples	Greece	Not specified	[39]
Performance	Moutelíková et al.	2018	Hospital-based	Czech Republic	Children	[40]
	Buss et al.	2015	Multicenter clinical study	USA	All ages	[41]
	Khare et al.	2014	Clinical laboratory	USA	All ages	[42]
	Wessels et al.	2014	Clinical laboratory	Netherlands	All ages	[43]
	Huang et al.	2016	Clinical laboratories	USA	All ages	[44]
	Coupland et al.	2013	Hospital-based	South Korea	All ages	[45]
	McAuliffe et al.	2013	Hospital-based	New Zealand	All ages	[46]
	Bennett et al.	2015	Hospital-based	Malawi	Children (< 5 years)	[47]
	Knoth et al.	2024	Multicenter clinical study	USA	All ages	[48]

### Molecular assays

Three sets of LAMP assays have been developed by Shuryaeva et al. (2022), Ziros et al. (2015) and Ye et al. (2018). These assays were designed for F40/F41 enteric adenoviruses [2] and rotavirus A [1], using primer sequences targeting the hexon region (for F40/F41 enteric adenoviruses) and segment 11 (for rotavirus A) (Supplementary data). These assays generally employed the standard LAMP primer design, consisting of four to six primers (F3, B3, FIP, BIP, and optional loop primers) recognizing six to eight distinct regions of the target sequence. Reaction conditions were typically performed at a constant temperature ranging from 60 °C to 65 °C for 30 to 60 min. Detection formats included colorimetric readouts, fluorescence-based detection, or turbidity measurement, allowing visual or instrument-assisted interpretation. Sample preparation requirements varied among studies, ranging from simple heat-treatment or crude extraction methods to conventional nucleic acid extraction kits, depending on the intended use and level of laboratory infrastructure. Twenty-six PCR assays were recorded, 17 of which were multiplex assays (capable of detecting many gastrointestinal pathogens (Table 2). Among the 26 PCR-based assays included, 6 were commercial platforms and 19 were laboratory-developed (in-house) assays. 12 of these assays used real-time PCR formats, reflecting the increasing adoption of quantitative and rapid detection technologies. The primers and probes used, and the regions targeted, are specified in the supplementary data.

**Table 2:** Molecular diagnostic assays used to detect Rotavirus and enteric adenoviruses F40/F41

Reference (Author, year)	Format	Virus target	Clinical validation
Allard et al., 2001	Laboratory-developed PCR	AdV	No
Avellón et al., 2001	Laboratory-developed PCR	AdV	No
Heim et al., 2003	Real-time PCR (laboratory-developed)	AdV	No
Pang et al., 2004	Real-time RT-PCR (laboratory-developed)	RVA	No
Logan et al., 2006	Real-time RT-PCR (laboratory-developed)	RVA, AdV	No
Gutiérrez-Aguirre et al., 2008	Real-time RT-PCR (laboratory-developed)	RVA	No
Nordgren et al., 2010	Real-time PCR (laboratory-developed)	RVA	No
Van Maarseveen et al., 2010	Multiplex real-time PCR (laboratory-developed)	RVA, AdV F	No
Khamrin et al., 2011	Multiplex PCR (laboratory-developed)	Enteric viruses	No
Liu et al., 2011	Luminex multiplex PCR (laboratory-developed)	RVA, AdV	No
Liu et al., 2012	Luminex multiplex PCR (laboratory-developed)	Enteric viruses	No
Liu et al., 2013	TaqMan Array Card (laboratory-developed)	Enteropathogens	No
Coupland et al., 2013	Commercial multiplex PCR (Seeplex^®^)	RV, AdV	Yes
McAuliffe et al., 2013	Multiplex PCR	Enteric viruses	Yes
Pang et al., 2014	Real-time PCR panel (laboratory-developed)	RVA	No
Wessels et al., 2014	Commercial platform (xTAG^®^ GPP)	RV, AdV	Yes
Khare et al., 2014	Commercial multiplex PCR	RV, AdV	Yes
Buss et al., 2015	Commercial platform (FilmArray^®^ GI)	RV, AdV	Yes
Bennett et al., 2015	Real-time PCR (laboratory-developed)	RVA	Yes
Huang et al., 2016	Comparative commercial panels	Enteric pathogens	Yes
Bennett & Gunson, 2017	Multiplex real-time PCR (laboratory-developed)	RV, AdV	No
Thongprachum et al., 2017	Multiplex RT-PCR (laboratory-developed)	Enteric viruses	No
Moutelíková et al., 2018	Real-time RT-PCR (laboratory-developed)	RVA	No
Kowada et al., 2018	Multiplex real-time PCR (laboratory-developed)	Enteric viruses	No
Ye et al., 2018	LAMP (laboratory-developed)	RVA	No
Mitra et al., 2020	Multiplex RT-PCR (laboratory-developed)	Enteric viruses	No
Knoth et al., 2024	Commercial multiplex PCR (BioCode^®^ GPP)	Enteric pathogens	Yes
Ziros et al., 2015	LAMP (laboratory-developed)	AdV F40/41	No
Shuryaeva et al., 2024	LAMP (laboratory-developed)	AdV F40/41	No

**LAMP**: Loop-mediated isothermal amplification; **RT-PCR**: Reverse-transcriptase polymerase chain reaction; **PCR**: Polymerase chain reaction; **AdV**: Adenovirus; **RV**: Rotavirus

### Performance of molecular diagnostic assays

The performance of the molecular diagnostic assays collected in this study was analyzed in terms of analytical sensitivity (expressed in copy/mL), clinical sensitivity and specificity (expressed as percentages) and test duration (expressed in minutes/seconds).

**Fig. 2:**
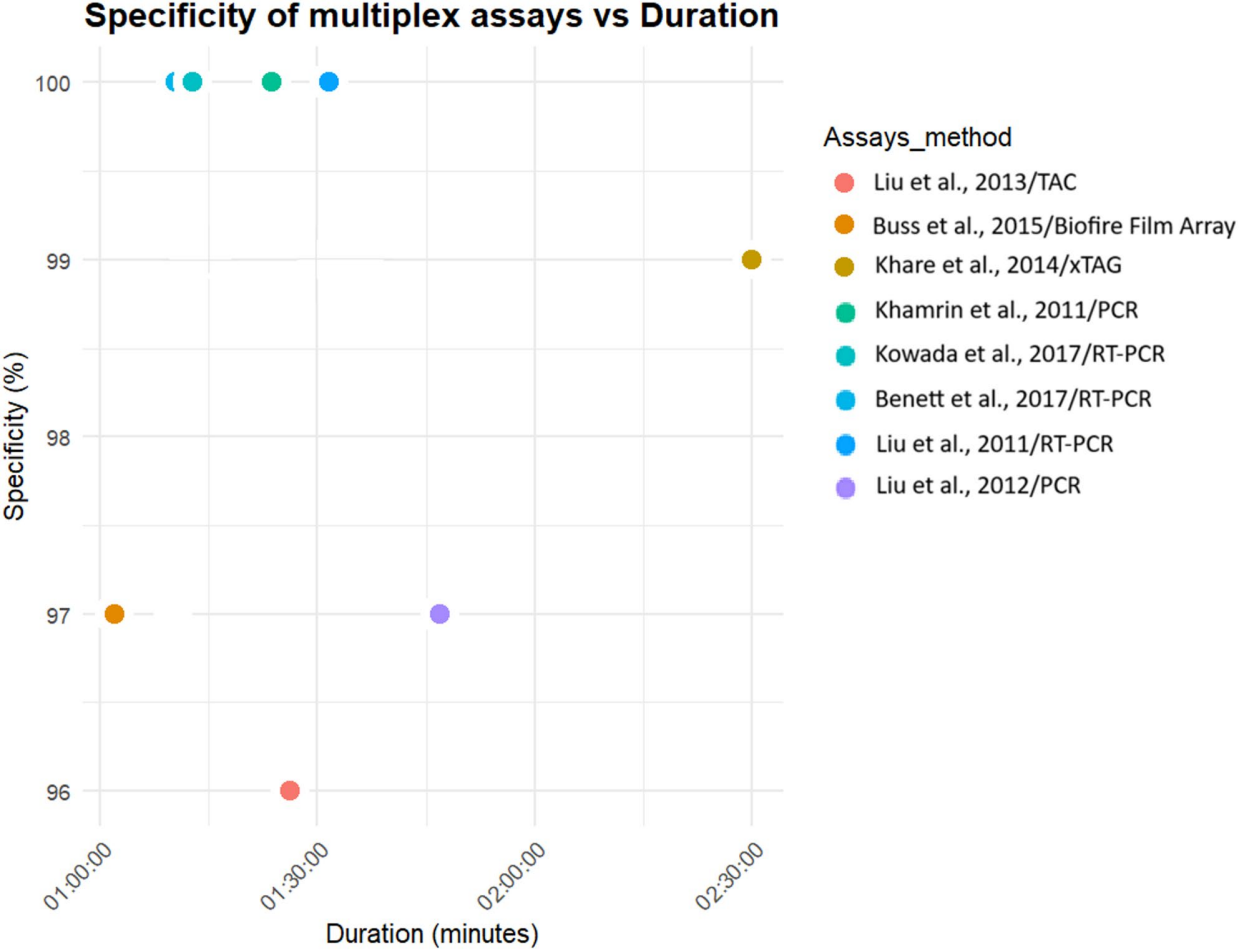
Specificity of multiplex molecular diagnostic assays

**Fig. 3:**
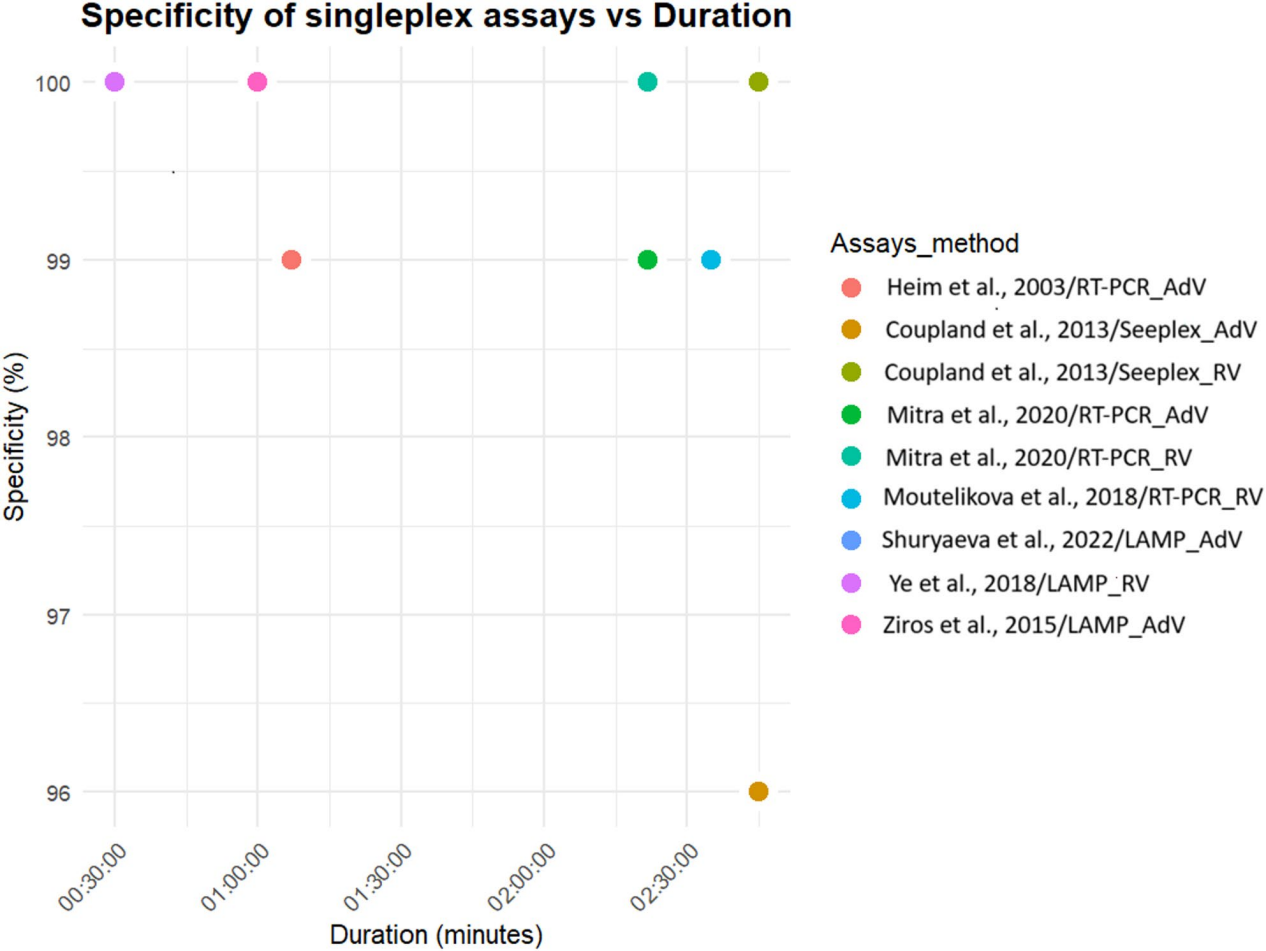
Specificity of singleplex molecular diagnostic assays. Shuryaeva et *al*. (2022) and Ye et *al*. (2018) have the same specificity and duration LAMP assays

Diagnostic specificity was assessed in 51% (15/29) of studies. This was generally assessed by testing with virus particles from similar viruses. Four sets of multiplex PCR assays [28, 29, 33, 35], three sets of singleplex LAMP assays [36, 38, 39] and two singleplex PCR assays [37, 45], show the highest specificities at 100% (Figs. 2 and 3).

Sensitivity assessment was carried out in 76% (22/29) of studies. Most often, a panel of positive samples was used, tested in parallel using alternative methods (cell culture, enzyme-linked immunosorbent assays, PCR). However, few studies explicitly stated whether the diagnostic evaluations were performed under blinded conditions, which may have introduced observer bias. In most cases, the same investigators conducted both the index and reference tests, suggesting that blinding was uncommon or unreported. PCR was most often used as the reference standard, but several studies did not clearly specify which test and/or clinical case definition was used as the reference standard. This approach presents methodological limitations. In several cases, PCR-based assays were evaluated against another PCR or RT-PCR method, rather than an independent diagnostic or clinical reference standard. This results in methodological circularity, where the index and reference tests share similar analytical principles and potential biases. Consequently, reported sensitivities and specificities may reflect analytical concordance rather than true diagnostic accuracy. Studies used composite reference standards, such as a combination of antigen detection (ELISA) or cell culture with PCR confirmation, which would have provided a more robust assessment of diagnostic validity.

**Fig. 4:**
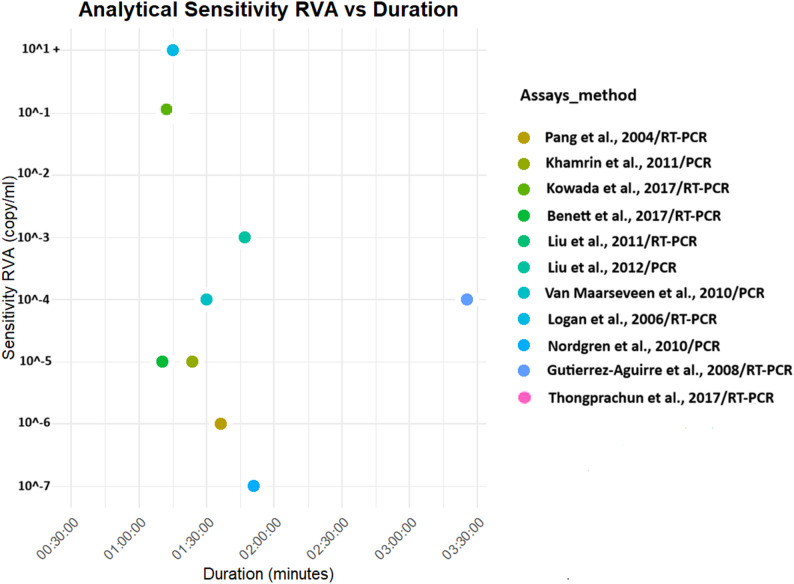
Analytical sensitivity of molecular diagnostics assays for Rotavirus A detection. Thongprachun et *al.* (2017), Liu et *al.* (2011) and Van Maarseven et *al*. (2010) have the same sensitivity and duration PCR assays

**Fig. 5:**
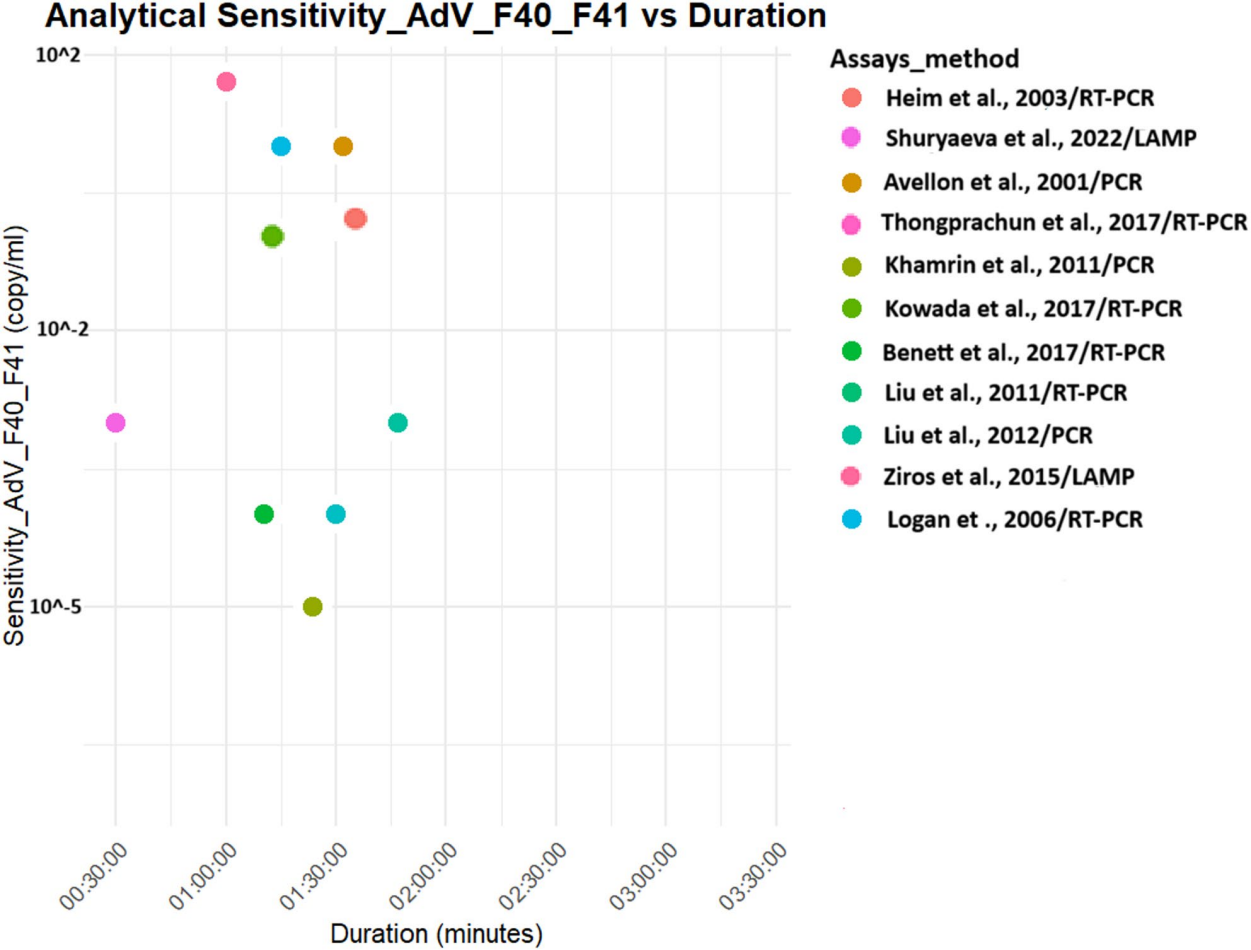
Analytical sensitivity of molecular diagnostics assays for Adenovirus F40/F41 detection. Thongprachun et *al.* (2017) and Liu et *al.* (2011) have the same sensitivity and duration PCR assays

**Fig. 6:**
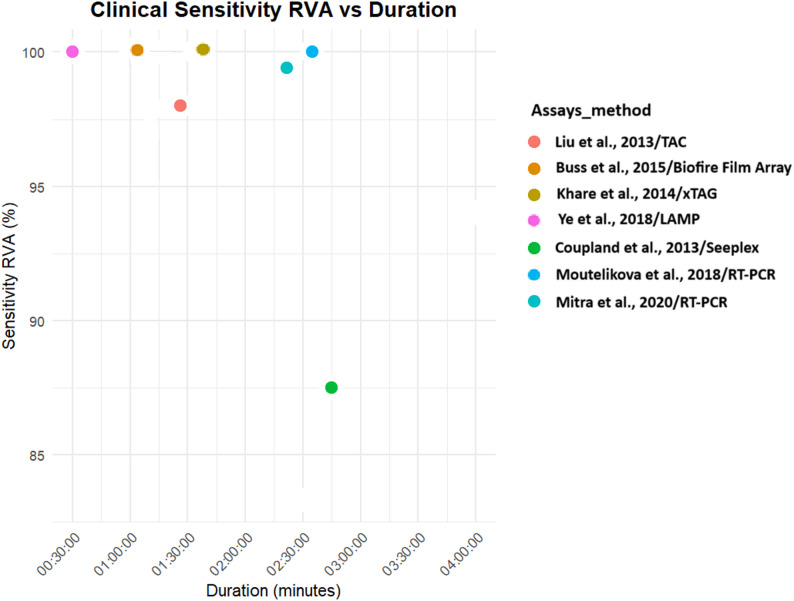
Clinical sensitivity of molecular diagnostics assays for Rotavirus A detection

**Fig. 7:**
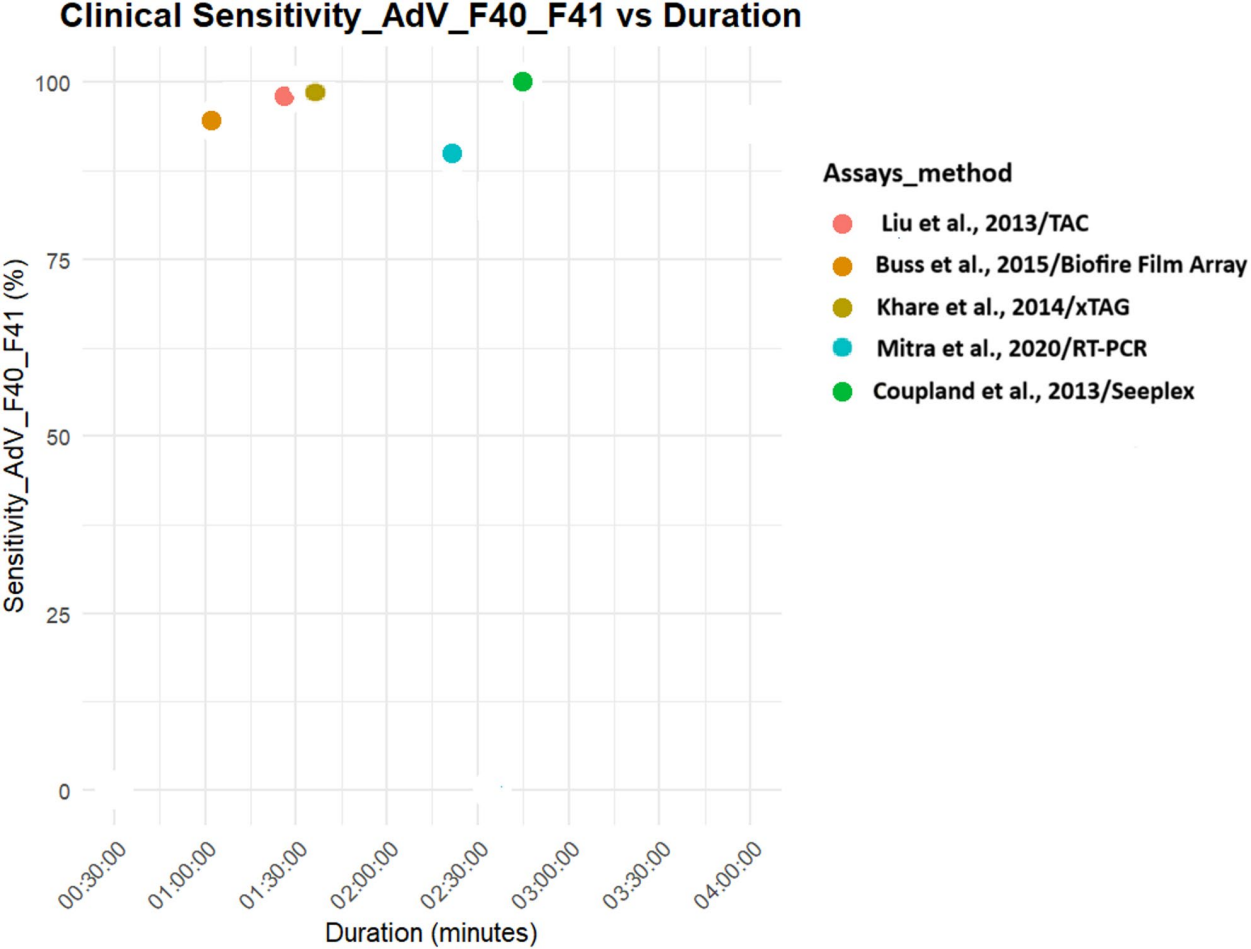
Clinical sensitivity of molecular diagnostics assays for Adenovirus F40/F41 detection

Both analytical and clinical sensitivities were higher for PCR tests [23, 27, 28, 33, 40–42, 45]. LAMP tests also gave significant positivity with a test duration that can facilitate point-of-care diagnosis [36, 38, 39] (Figs. 4, 5, 6 and 7).

Among the molecular diagnostic assays reviewed, three PCR assays [28, 33, 41] and three LAMP assays [36, 38, 39] fulfilled point-of-care criteria, demonstrating very good performance overall particularly the LAMP assays, which stood out for their simplicity and rapid turnaround (Table 3).

**Table 3:** Best point-of-care molecular diagnostic assays

Assays method	Se RVA	Se AdV F40/F41	Specificity	Duration
Benett et *al*., 2017/RT-PCR	10^-5 copy/mL	10^-4 copy/mL	100%	~ 70 min
Khamrin et *al*., 2011/PCR	10^-5 copy/mL	10^-5 copy/mL	100%	~ 1 h 23 min
Buss et *al*., 2015/Biofire Film Array	100%	94,5%	97,1%	1 h
Shuryaeva et al.,2022/LAMP	-	10^-3 copy/mL	100%	30 min
Ziros et al., 2015/LAMP	-	50–100 copy/mL	100%	60 min
Ye et al., 2018/LAMP	100%	-	100%	30 min

**Se**: sensitivity

For several assays, only amplification time or system run time was reported, without clear distinction between sample preparation, nucleic acid extraction, amplification, and result readout. For example, LAMP assays reported a 30–60 min amplification time, while PCR assays reported total system run times of approximately 1–2 h without specifying individual workflow steps. This lack of detail may underestimate the true turnaround time in point-of-care settings.

## Discussion

Recent molecular epidemiological studies reinforce the importance of sensitive diagnostic tools for enteric viruses. For instance, a 2026 study in Iraqi children reported that rotavirus remained the predominant viral pathogen, with a detection rate of 45% and notable co-infection patterns with adenovirus and norovirus [49]. Regional reviews have also demonstrated the genetic diversity and continued circulation of rotavirus strains, highlighting the importance of molecular surveillance to guide both vaccine strategies and diagnostic development [50]. Such findings highlight the limitations of single-target diagnostic approaches and support the development of multiplex molecular assays suitable for point-of-need testing. Furthermore, recent work on diarrheic virus-host interactions has demonstrated the complex interplay between viral infection, host immunity, and the gut microbiome, emphasizing the need for precise and timely diagnostics to better understand disease mechanisms and guide clinical management [51].

A rapid and sensitive test is a crucial tool in the implementation of a systematic response to infectious diseases [52]. In this respect, the high throughput, sensitivity and specificity of molecular assays enable rapid diagnosis and improved management of gastrointestinal infections [32]. Point-of-care molecular tests have also been developed, which are more accessible and less resource-intensive, facilitating monitoring at all levels.

This review included 29 studies describing PCR and LAMP assays capable of detecting rotavirus and enteric adenovirus F40/F41. The majority of tests were evaluated in terms of analytical and clinical performance. However, studies differed in design, reaction mixtures (in addition to primers and probes), melting temperature, incubation time, and some omitted key descriptive features and experimental details. Such heterogeneity and incomplete reporting limit the comparability and reproducibility of results across studies. Adherence to standardized reporting frameworks such as the STARD (Standards for Reporting Diagnostic Accuracy Studies) guidelines would help improve transparency and allow more meaningful synthesis in future systematic reviews [53]. These guidelines recommend clear documentation of study design, participant selection, index and reference test procedures, and statistical analyses, thereby enhancing the interpretability of diagnostic performance data. In this review, the classification of assays as point-of-care was based not only on diagnostic accuracy but also on operational characteristics such as turnaround time, equipment requirements, and suitability for decentralized use, which are critical factors in low-resource and outbreak settings [54].

The results of our systematic review show an increasing development of molecular diagnostic assays for the detection of rotavirus and enteric adenoviruses F40/F41 responsible for most gastroenteritis, especially in children under 5 years. These molecular assays play an important role in monitoring these two viruses worldwide, despite the inaccessibility of some of them in remote areas.

Among the molecular diagnostic assays included in this review, three PCR assays [28, 33, 41] and three LAMP assays [36, 38, 39] meet the point-of-care criteria with satisfactory performances, especially the LAMP assays [55], which are potentially applicable as a bedside diagnostic method, requiring no specific instruments and providing rapid results [56]. PCR methods have two significant drawbacks: expensive equipment and the time required to perform the test (between 120 and 150 min for most PCR assays), which limits its application in field situations and in small clinical laboratories lacking the appropriate equipment [57].

The performance of the included molecular diagnostic assays was assessed on the basis of their specificity, sensitivity and test duration. Overall, the specificity was good (96 to 100%), with virtually no incidents of non-specific amplification reported by subsequent studies using the same tests. The specificity of LAMP assays was 100% for all three assays due to the use of six to nine primers, with two loop primers recognizing eight distinct regions on the target sequence [39]. However, we noted that the use of different primer sets for RT-PCR could lead to different performances. In clinical laboratories, polymerase chain reaction (PCR)-based assays are considered as gold-standard for the detection of viruses, but when it comes to multiplex detections, variations in the properties of viral nucleic acids could reduce the specificity [58, 59].

The sensitivity of the molecular diagnostic assays included in this study is also high in several PCR assays [23, 27, 28, 33, 40–42, 45] and 100% of LAMP assays [36, 38, 39]. Real-time RT-PCR proved far more sensitive than other methods, with analytical detection limits 1000 times lower than those of conventional RT-PCR and 100 times lower than those of conventional nested PCR [60]. The single-step RT-PCR method included in this review uses a closed-tube system, reducing the risk of contamination and providing results within a few hours [29]. This leads to efficient detection, as viruses have been identified in a substantial proportion of outbreaks where environmental surfaces have also been tested for viral presence [60]. Thus, molecular diagnostics assays, particularly for rotavirus and enteric adenovirus F40/F41, play an essential role in the identification of viral agents in cases of gastroenteritis in young children, offering high sensitivity for pathogen detection. However, RT-PCR exhibits some limitations such as the fact that they are not offering a point-of-care solution, require considerable resources and long test times (with the exception of [28]; [33], [41]). However, RT-PCR has important limitations, as it is not suitable for point-of-care use and requires specialized equipment, trained personnel, and relatively long turnaround times.

Molecular diagnostic assays using LAMP to detect viruses such as rotavirus and enteric adenoviruses F40/F41 are increasingly used due to their specificity and sensitivity. In addition, the mean test duration identified for these assays included in this review exhibited that they could be useful for point-of-care diagnosis as previously described for the detection of enteric adenoviruses F40/F41 [38, 39] and rotavirus A [36]. These LAMP assays have shown high sensitivity in fecal samples, which holds promise for rapid and accurate point-of-care applications without the need for sophisticated laboratory equipment, particularly in resource-limited settings. Overall, the results of this systematic review show that LAMP assays specific to rotavirus A and enteric adenovirus F40/F41 exhibited high performances. Combined with new sample preparation and detection methods, these assays could be useful as portable molecular diagnostic tools, particularly in regions where these two gastrointestinal viruses are endemic.

This systematic review has several limitations related both to the included studies and to the review process itself. First, the primary studies showed substantial methodological heterogeneity. The assays differed in target genes, amplification formats, sample preparation steps, and reference standards, which limited direct comparison of performance indicators. In addition, several studies reported incomplete diagnostic accuracy data, and blinding procedures were rarely described, suggesting a potential risk of diagnostic bias. Second, some limitations are inherent to the review methodology. The search strategy, although comprehensive, yielded a very large number of records, which may reflect non-specific search terms. Only selected databases were included, and language restrictions may have excluded relevant studies. Furthermore, a formal risk-of-bias assessment was not conducted, and no quantitative meta-analysis was performed because of the heterogeneity of study designs and reported outcomes. An additional limitation of the included studies was the limited reporting of blinding procedures. In most studies, it was unclear whether index test interpretation was performed independently of reference test results, which may have introduced diagnostic bias.

These factors should be considered when interpreting the findings, and they may affect the generalizability of the conclusions. Future systematic reviews should incorporate standardized reporting criteria, formal risk-of-bias tools, and meta-analytic methods where appropriate.

## Conclusion

Beyond analytical performance, accurate molecular diagnostics have important implications for both clinical management and public health. Rapid and sensitive detection of rotavirus and enteric adenoviruses can support timely clinical decision-making, reduce unnecessary antibiotic use, and improve patient triage, particularly in pediatric settings. At the population level, reliable molecular diagnostic tools play a critical role in surveillance systems by enabling accurate case identification, monitoring of circulating genotypes, and early detection of outbreaks. Such tools are also essential for evaluating vaccine impact and detecting potential genotype shifts following vaccine introduction. In this context, point-of-care molecular diagnostics could significantly enhance both patient care and public health responses, especially in resource-limited settings where laboratory infrastructure is limited and the burden of diarrheal diseases remains high.

## Supplementary Information

Below is the link to the electronic supplementary material.

Supplementary Material 1

## Data Availability

All data generated or analyzed during this study are included in this published article and its supplementary information files.

## Abbreviations

PRISMA: Preferred Reporting Items for Systematic reviews and Meta-Analyses
LAMP: Loop-mediated isothermal amplification
PCR: Polymerase chain reaction
HAdV: Human adenovirus
WHO: World Health Organization
ICTV: International Committee on Taxonomy of Viruses
PMC: PubMed Central
EIA: Enzyme immunoassays
ELISA: Enzyme-linked immunosorbent assays
CLIA: Clinical Laboratory Improvement Amendments
STARD: Standards for Reporting Diagnostic Accuracy Studies
