# Supplementary material for: Systematic review of point-of-need molecular diagnostics for rotavirus and enteric adenoviruses F40/F41
Source: BMC Infect Dis. 2026 Mar 13;26:792. doi: 10.1186/s12879-026-13081-4 (PMC13097646; doi:10.1186/s12879-026-13081-4)
Supplement: Supplementary file 1 — Supplementary Material 1 [file 12879_2026_13081_MOESM1_ESM.docx]

**Supplementary data**

**Table 4:** Primers and target region of the molecular diagnostic assays collected in this study

| **Primers Rotavirus A** | **Target region** | **Primers Enteric adenoviruses F40/F41** | **Target region** | **References** |
| --- | --- | --- | --- | --- |
| F- ACC ATC TAC ACA TGA CCC TC | NSP3 | F -GCC ACG GTG GGG TTT CTA AAC TT | Hexon | Benett *et al*., 2017 |
| R- GGT CAC ATA ACG CCC C |  | R -GCC CCA GTG GTC TTA CAT GCA CAT C |  |  |
| FAM- ATG AGC ACA ATA GTT AAA AGC TAA CAC TGT CAA |  | Atto647 TGC ACC AGA CCC GGG CTC AGG TAC TCC GA |  |  |
|  |  | hex1deg (5′-GCC SCA RTG GKC WTA CAT GCA CAT C-3 ') | Hexon | Allard *et al*., 2001 |
|  |  | hex2deg (5′-CAG CAC SCC ICG RAT GTC AAA-3') |  |  |
|  |  | nehex3deg (5′-GCC CGY GCM ACI GAI ACS TAC TTC-3 ') |  |  |
|  |  | nehex4deg (5′-CCY ACR GCC AGI GTR WAI CGM RCY TTG TA-3') |  |  |
| F, ACCATCTWCACRTRACCCTCTATGAG | NSP3 | F, GCCACGGTGGGGTTTCTAAACTT | Hexon | Liu *et al*., 2013 |
| R, GGTCACATAACGCCCCTATAGC |  | R, GCCCCAGTGGTCTTACATGCACATC |  |  |
| P, AGTTAAAAGCTAACACTGTCAAA |  | P, TGCACCAGACCCGGGCTCAG |  |  |
|  |  | **FOP AdvF3.1 TGTATGCGCCTCCTGTGTTA** | **Hexon** | **Shuryaeva *et al*., 2022** |
|  |  | **FOP AdvF3.2 TGTGTACGCCTCCTGTGTTA** |  |  |
|  |  | **ROP AdvВ3 ACRAAKCGCAGCGTCAGTC** |  |  |
|  |  | **LP AdvFL GCATGTAAGACCATTGCGGCATCA** |  |  |
|  |  | **LP AdvBL ACSTACTTCAGCCTGGGGAACAA** |  |  |
|  |  | **FIP AdvFIP ACCAGGCCCGGRCTCAGRTATTTTGCCAGASAGCCGAGTGAC** |  |  |
|  |  | **RIP AdvBIP1 CARTTTGCCCGCGCCACCGATTTTTTACATCGTGGGTSGGAGC** |  |  |
|  |  | **RIP AdvBIP2 CAGTTCGCCCGTGCCACCGATTTTTTACATCGTGGGTSGGAGC** |  |  |
|  |  | **Probe/LP AdvFL.NatTail R6G NatTail-GCATGTAAGACCATTGCGGCATCAb** |  |  |
|  |  | **Quencher FLQ Common quencher -BHQ1b** |  |  |
| ACCATCTWCACRTRACCCTC | NSP3 |  |  | Moutelikova *et al.*, 2018 |
| CACATAACGCCCCYATAGCC |  |  |  |  |
| ATGAGCACAATAGTTAAAAGCTAACACTGTCAA |  |  |  |  |
| JVKF- CAGTGGTTGATGCTCAAGATGGA | NSP3 | JTVXF- GGACGCCTCGGAGTACCTGAG | Hexon | Kowada *et al*., 2017 |
| JVKR- TCATTGTAATCATATTGAATACCCA |  | JTVXR- ACIGTGGGGTTTCTGAACTTGTT |  |  |
| JVKP- ACAACTGCAGCTTCAAAAGAAGWGT |  | JTVXP- CTGGTGCAGTTCGCCCGTGCCA |  |  |
| VP2-F1 TCTGCAGACAGTTGAACCTATTAA | VP2 |  |  | Gutierrez-Aguirre *et al.*, 2008 |
| VP2- F2a CAGACACGGTTGAACCCATTAA |  |  |  |  |
| VP2- F3a TCGGCTGATACAGTAGAACCTATAAATG |  |  |  |  |
| VP2- F4a TGTCAGTGATACAGTAGAACCTATAAATG |  |  |  |  |
| VP2- F5a TCAGCTGACACAGTAGAACCTATAAATG |  |  |  |  |
| VP2-R1un GTTGGCGTTTACAGTTCGTTCAT |  |  |  |  |
| VP2- R2a GTTGGCGTCTACAATTCGTTCAT |  |  |  |  |
| VP2- Pb 6FAM-ATGCGCATRTTRTCAAAHGCAA |  |  |  |  |
| sBeg9 GGCTTTAAAAGAGAGAATTTC | VP7 | Ad1 TTCCCCATGGCICAYAACAC | Hexon | Thongprachun *et al.*, 2016 |
| VP7-1′ ACTGATCCTGTTGGCCATCCTTT |  | Ad2 CCCTGGTAKCCRATRTTGTA |  |  |
|  |  | **F3_ADE CCGCAATGGTCTTACATGCA** | **Hexon** | **Ziros *et al.*, 2015** |
|  |  | **B3_ADE GAATAAGCGGTGTCCTCGC** |  |  |
|  |  | **FIP_ADE CTGAAGTACGTATCGGTGGCGCGACGCCTCGGAGTATCTGAG** |  |  |
|  |  | **BIP_ADE TCAGAAATCCCACTGTGGCTCCCGAATCGCAGCGTCAGTC** |  |  |
|  |  | **LF_ADE AATTGCACCAGGCCCGG** |  |  |
|  |  | **LB_ADE CACGATGTAACCACAGACAGGTC** |  |  |
| AACCATCTACACATGACCCTCTATGA | NSP3 | GCCACGGTGGGGTTTCTAAACTT | Hexon | Pang *et al*., 2004; Heim *et al*., 2003 |
| GGTCACATAACGCCCCTATAGC |  | GCCCCAGTGGTCTTACATGCACATC |  |  |
| CAATAGTTAAAAGCTAACACTGTCAAA |  | TGCACCAGACCCGGGCTCAGGTACTCCGA |  |  |
| ACC ATC TTC ACG TAA CCC TC | NSP3 | 5'-GCC-ACG-GTG-GGG-TTT-CTA-AAC-TT-3' | Hexon | Van Maarsevven *et al*., 2010; Heim *et al*., 2003 |
| ACC ATC TAC ACA TGA CCC TC |  |  |  |  |
| CAC ATA ACG CCC CTA TAG CC |  | 5'-GCC-CCA-GTG-GTC-TTA-CAT-GCA-CAT-C-3' |  |  |
| TXR-ATGAGCACAATAGTTAAAAGCTAACACTGTCAA-BHQ2 |  | 5'-TGC-ACC-AGA-CCC-GGG-CTC-AGG-TAC-TCC-GA-3' |  |  |
| TTC CAC CAG GYA TGA ATT GGA C | VP6 |  |  | Nordgren *et al.*, 2010 |
| catgtGGT CCT CAC TTA ATC AAC A t G |  |  |  |  |
| cttgtCTT GGT CCT CAT TTG A  cA AG |  |  |  |  |
| AGGTGACACTATAGAATA AAGTCTCRACATGGAKGT | VP6 | AGGTGACACTATAGAATA AGACAGGTCACAGCGACTGA | Hexon | Liu *et al.*, 2012 |
| GTACGACTCACTATAGGGA ARRTTICCAATTCCTCCAGT |  | GTACGACTCACTATAGGGA TTATAGGCGGTTCCGGAGTA |  |  |
| ATAGTAACCATGAACGGAAA |  | GCACGAATCGCAGCGTCAGT |  |  |
| VP7 1 (F) AAA GGA TGG CCA ACA GGA TCA GT | VP7 | Ad1 TTC CCC ATG GCI CAY AAC AC | Hexon | Khamrin *et al.*, 2011 |
| End9(s) GTA TAR AAH ACT TGC CAC CAT |  | Ad2 CCC TGG TAK CCR ATR TTG TA |  |  |
| ACCATCTWCACRTRACCCTCTATGAG | NSP3 | GCCACRGTGGGRTTTCTCAACTT | Hexon | Liu *et al*., 2011 |
| GGTCACATAACGCCCCTATAGC |  | GCCGCAATGGTCTTACATGCACATC |  |  |
| AGTTAAAAGCTAACACTGTCAAA |  | TGCACCAGGCCCGGGCTCAG |  |  |
|  |  | 5'-GCC-ACG-GTG-GGG-TTT-CTA-AAC-TT-3' | Hexon | Heim *et al.*, 2003 |
|  |  | 5'-GCC-CCA-GTG-GTC-TTA-CAT-GCA-CAT-C-3' |  |  |
|  |  | 5'-TGC-ACC-AGA-CCC-GGG-CTC-AGG-TAC-TCC-GA-3' |  |  |
| NVP3-F1 ACCATCTACACATGACCCTC | NSP3 | Ead-F CCGACCCACGATGTAACCA | Hexon | Pang *et al.*, 2014; 2011 |
| **F3, CTACAACGTCAACTCTTTCTG** | **Segment 11** |  |  | **Ye *et al*., 2018** |
| **B3, AATCCATAGACACGCCAG** |  |  |  |  |
| **FIP, CGACAACATGTACTTATTGAATGCCAAAATCTATTGGTAGGAGTGAACA** |  |  |  |  |
| **BIP, TCTCCAGAGGATATTGGACCATCTTGTCTTAACTGCATTCGATCT** |  |  |  |  |
| **LF, TCTGCATCCGGTGAAATGTA** |  |  |  |  |
| **LB, AACGATCCACTCACCAGCTTTTCGA** |  |  |  |  |
| ROTAs ACC ATC TTC ACG TAA CCC TC | NSP3 | ADVs CAT GAC TTT TGA GGT GGA TC | Hexon | Van Maarseveen *et al*., 2010 |
|  |  | ADVas CCG GCC GAG AAG GGT GTG CGC AGG TA |  |  |
| ROTAs ACC ATC TAC ACA TGA CCC TC |  | ADVs TAT GAC ATT TGA AGT TGA CC |  |  |
|  |  | ADV-MGB-FAM 6FAM-AGC CCA CCC TKC TTT AT-MGBNFQ |  |  |
| ROTAas CAC ATA ACG CCC CTA TAG C |  | ADVFs CTC GAC ATG ACT TTT GAG GT |  |  |
|  |  | ADVFas GTA GAC GGC CTC GAT GAC |  |  |
| ROTA-TQ-TXR TXR-ATGAGCACAATAGTTAAAAGCTAACACTGTCAA-BHQ2 |  | ADVF-MGB-FAM 6FAM-AGC CCA CAC TTC T-MGBNFQ |  |  |
| RotaA-fwd1 GGATGTCCTGTACTCCTTGTCAAAA | VP6 | Adeno.fwd TTCCAGCATAATAACTCWGGCTTTG | Hexon | Logan *et al*., 2006 |
| RotaA-fwd2 GGAGGTTCTGTACTCATTGTCAAAAA |  |  |  |  |
| RotaA.rev1 TCCAGTTTGGAACTCATTTCCA |  | Adeno.rev AATTTTTTCTGWGTCAGGCTTGG |  |  |
| RotaA.rev2 TCCAGTTTGAAAGTCATTTCCATT |  |  |  |  |
| RotaA.probe1 (VIC) ATAATGTGCCTTCGACAAT |  | Adeno.probe1 (FAM) CCATACCCCCTTATTGG |  |  |
| RotaA.probe2 (VIC) AATATAATGTACCTTCAACAAT |  | Adeno.probe2 (FAM) CCTTACCCCCTTATTGG |  |  |
| RV VP6-F 5′-GACGGVGCRACTACATGGT-3′ | VP6 | AdV-F 5′-AGATGAGAATGCTGCCGGA-3′ | E1A protein | Mitra *et al.*, 2020 |
| RV VP6-R 5′-GTCCAATTCATNCCTGGTG-3′ |  | AdV-R 5′-CAACCCCAGAATCRCCCGAA-3′ |  |  |

LAMP assays are shown in bold
